# Mesenchymal stem cell bioenergetics and apoptosis are associated with risk for bronchopulmonary dysplasia in extremely low birth weight infants

**DOI:** 10.1038/s41598-022-22478-5

**Published:** 2022-10-19

**Authors:** Snehashis Hazra, Rui Li, Bianca M. Vamesu, Tamas Jilling, Scott W. Ballinger, Namasivayam Ambalavanan, Jegen Kandasamy

**Affiliations:** 1 Department of Pediatrics, University of Alabama at Birmingham School of Medicine, 1700 6th Avenue South, Birmingham, AL 35233 USA; 2 Department of Pathology, University of Alabama at Birmingham School of Medicine, Birmingham, USA

**Keywords:** Mitochondria, Mesenchymal stem cells, Bioenergetics, Respiratory tract diseases, Neonatology, Paediatrics, Preterm birth

## Abstract

Oxidant stress contributes significantly to the pathogenesis of bronchopulmonary dysplasia (BPD) in extremely low birth weight (ELBW) infants. Mitochondrial function regulates oxidant stress responses as well as pluripotency and regenerative ability of mesenchymal stem cells (MSCs) which are critical mediators of lung development. This study was conducted to test whether differences in endogenous MSC mitochondrial bioenergetics, proliferation and survival are associated with BPD risk in ELBW infants. Umbilical cord-derived MSCs of ELBW infants who later died or developed moderate/severe BPD had lower oxygen consumption and aconitase activity but higher extracellular acidification—indicative of mitochondrial dysfunction and increased oxidant stress—when compared to MSCs from infants who survived with no/mild BPD. Hyperoxia-exposed MSCs from infants who died or developed moderate/severe BPD also had lower PINK1 expression but higher TOM20 expression and numbers of mitochondria/cell, indicating that these cells had decreased mitophagy. Finally, these MSCs were also noted to proliferate at lower rates but undergo more apoptosis in cell cultures when compared to MSCs from infants who survived with no/mild BPD. These results indicate that mitochondrial bioenergetic dysfunction and mitophagy deficit induced by oxidant stress may lead to depletion of the endogenous MSC pool and subsequent disruption of lung development in ELBW infants at increased risk for BPD.

## Introduction

Supplemental oxygen frequently required for prolonged periods (hyperoxia) by prematurely born extremely low birth weight (ELBW) infants contributes to oxidant stress in their lungs that would otherwise develop in a hypoxemic intrauterine environment. Such oxidant stress is a major contributor to the pathogenesis of bronchopulmonary dysplasia (BPD) which is characterized by disrupted pulmonary alveolar and vascular development and remains the leading cause of morbidity and mortality in up to 60% of ELBW infants despite improved neonatal intensive care^1–3^. Therefore, there is a critical need to identify new biomarkers and therapeutic targets to decrease oxidant stress, neonatal lung injury and BPD risk in ELBW infants.

Oxidant stress can induce apoptotic cell death through release of mitochondrial cytochrome c. Mitochondria are also major sources of reactive oxygen species (ROS), and mitochondrial electron transport chain (ETC) bioenergetic function as well as mitochondrial DNA (mtDNA) are known to be highly sensitive to oxidant injury. Consequently, mitochondrial function and quality control mechanisms such as PINK1 (PTEN-induced putative kinase 1) mediated mitophagy that removes dysfunctional mitochondria play a critical role in maintaining cellular homeostasis during oxidant stress^4–7^. Our previous studies indicate that mitochondrial dysfunction is associated with neonatal lung injury and increased BPD risk in ELBW infants^8,9^. Other studies suggest that insufficient or unregulated mitophagy may increase susceptibility to lung diseases such as chronic obstructive pulmonary disease and pulmonary hypertension^10–12^. Consequently, studying the association between oxidant stress-induced mitochondrial dysfunction and dysregulated mitophagy, metabolism and survival in cells critical for early lung development could help identify novel mechanisms for BPD pathogenesis in ELBW infants.

In this context, cellular bioenergetics and redox state have been known to regulate self-renewal and differentiation of mesenchymal stem cells (MSCs) that contribute significantly to fetal and neonatal lung development. MSC derived extracellular vesicles (EVs) that mediate their paracrine anti-inflammatory and angiogenic effects also contain functional mitochondria that can modify their activity and uptake^13^. MSCs have also been known to transfer mitochondria to restore bioenergetic function in alveolar and airway epithelial cells in models of acute lung injury and asthma^14–17^. MSC injury repair ability is reduced by adverse local microenvironments that overwhelm their autophagy and mitophagy processes and trigger apoptosis^18–20^. However, whether mitochondrial function and autophagy efficiency of endogenously available MSCs are associated with their proliferative ability and survival as well as BPD severity in ELBW infants remains unknown. While performing such studies using lung tissue-resident MSCs could help improve tissue specificity, access to such cells from ELBW infants is limited, making umbilical cord-derived MSCs that are from a distant biological site but easier to obtain a more viable alternative.

Therefore, in this study we tested the hypothesis that MSCs derived from umbilical cords of ELBW infants who die or develop moderate/severe BPD have decreased mitochondrial bioenergetics, and impaired autophagy/mitophagy, proliferation and survival when compared to MSCs from survivors with no/mild BPD.

## Results

### Baseline characteristics

39 ELBW infants were enrolled in the study and followed to determine their BPD status and severity using NICHD consensus guidelines. 18 of these infants survived to discharge with no/mild BPD whereas 21 infants developed moderate/severe BPD or died before 36 weeks post-menstrual age (PMA). Known BPD risk factors such as birth weight, gestational age, gender, ethnicity, intrauterine growth restriction (IUGR), maternal preeclampsia and chorioamnionitis (inflammation of placental membranes) were also compared between the two groups. As expected, infants who died or developed moderate/severe BPD had lower BW compared to infants who survived with no/mild BPD. Caucasian infants and infants without exposure to maternal chorioamnionitis had a higher incidence of moderate/severe BPD or death before discharge compared to African-American (AA) infants and infants exposed to maternal chorioamnionitis. However, median GA, IUGR incidence, sex and exposure to maternal preeclampsia did not differ significantly between infants who died or developed moderate/severe BPD and survivors with no/mild BPD (Table 1).

**Table 1:** Clinical characteristics and MSC bioenergetics by BPD Status.

Characteristic	N	None/mild, N = 18^1^	Mod/sev, N = 21^1^	*p* value^2^
GA (weeks)	39	26.00 [25.25, 27.00]	25.00 [24.00, 26.00]	0.2
Birth weight (g)	39	870 [704, 980]	690 [595, 840]	**0.036**
Sex (female)	39	10 [56%]	9 [43%]	0.4
Intrauterine growth restriction	39	3 [17%]	5 [24%]	0.7
Ethnicity	39			**0.002**
Caucasian		3 [17%]	14 [67%]	
African-American		15 [83%]	7 [33%]	
Histologic chorioamnionitis	39	17 [94%]	2 [9.5%]	** < 0.001**
Preeclampsia/PIH/eclampsia	39	5 [28%]	8 [38%]	0.5
Maternal smoking	39	7 [39%]	7 [33%]	0.7
Basal OCR (pmol/min/30 k cells)	29	67 [47, 98]	36 [27, 44]	** < 0.001**
ATP-linked OCR (pmol/min/30 k cells)	29	36 [28, 45]	14 [12, 21]	** < 0.001**
Non-mitochondrial OCR (pmol/min/30 k cells)	29	11 [9, 22]	16 [11, 21]	0.5
Leak OCR (pmol/min/30 k cells)	29	16 [9, 25]	5 [4, 7]	** < 0.001**
Maximal OCR (pmol/min/30 k cells)	29	143 [101, 209]	73 [70, 82]	** < 0.001**
Spare respiratory capacity (pmol/min/30 k cells)	29	62 [45, 99]	37 [32, 46]	**0.003**
Complex IV OCR (pmol/min/30 k cells)	29	125 [79, 170]	63 [30, 93]	**0.023**
MSC ATP content (pMol/cell)	29	29.82 [26.81, 30.21]	25.23 [23.70, 26.70]	** < 0.001**
Basal ECAR (mpH/min/30 k cells)	29	37 [36, 45]	52 [46, 60]	** < 0.001**
Glycolytic capacity (mpH/min/30 k cells)	29	56 [45, 66]	82 [61, 95]	**0.004**

^1^Median [IQR]; n [%] ^2^Student's t-test; Pearson's Chi-squared test; Wilcoxon rank sum exact test.

Significant values are in [bold].

### MSC bioenergetic dysfunction is higher in ELBW infants with moderate/severe BPD

**Figure 1:**
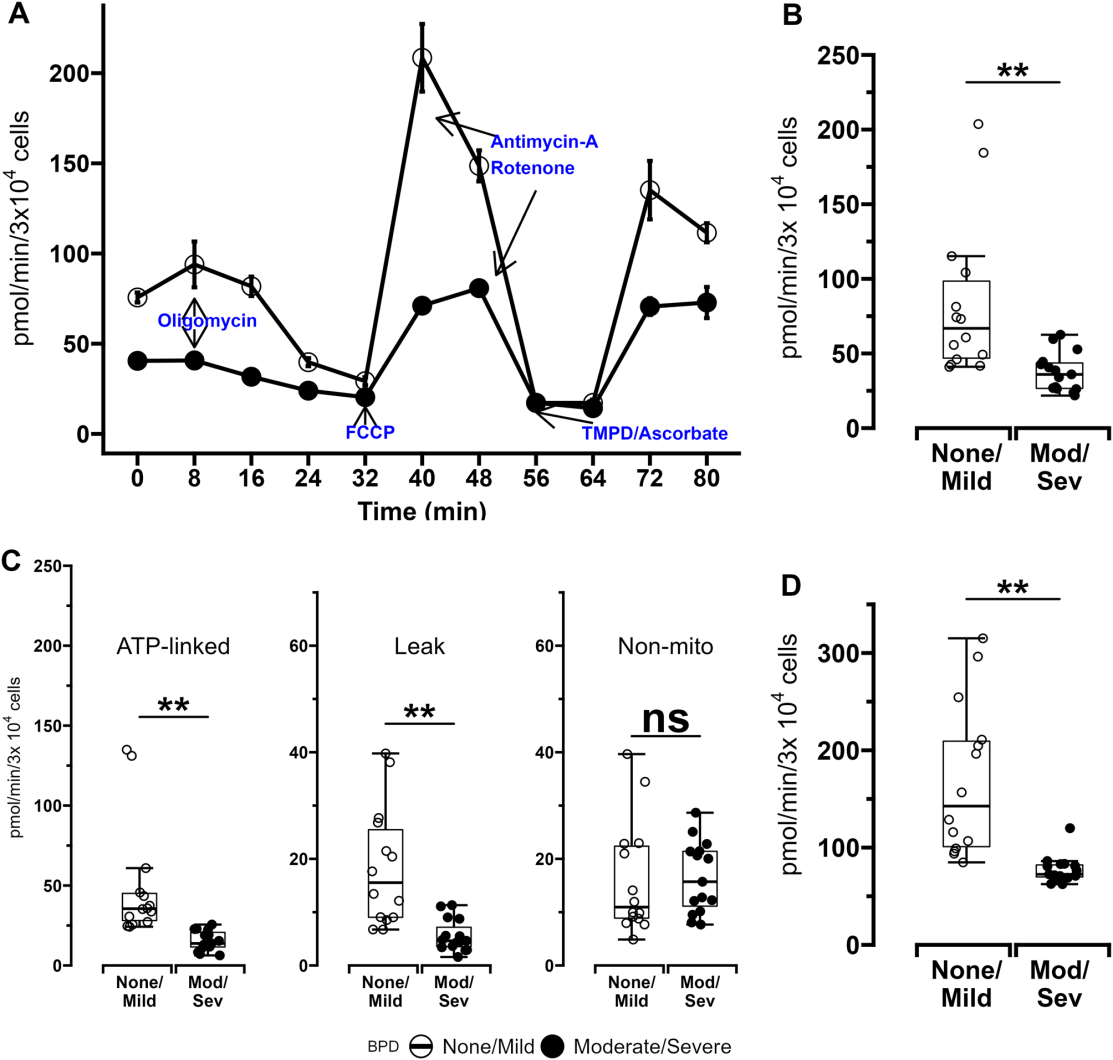
Oxygen consumption rates (OCR) of MSCs obtained from ELBW infants who died or developed moderate/severe BPD (Mod/Sev) and ELBW infants who survived with no/mild BPD (None/Mild). (**A**) Representative plot of a typical mitochondrial stress test conducted using a Seahorse XF96 flux analyzer. 30,000 cells were seeded per well in 96-well plates, and MSC oxygen consumption was measured in the presence of various mitochondrial effectors. (**B**) Basal OCR. (**C**) Panel with plots showing MSC ATP-linked OCR, proton leak, and non-mitochondrial OCR. (**D**) Maximal OCR measured after FCCP introduction. Data for all experiments obtained from MSCs from 15 infants with Mod/Sev BPD and 14 with No/Mild BPD. Differences were analyzed using Mann–Whitney U-test and data expressed as median [25th–75th centiles]. * and ** represent *p* value < 0.05 and < 0.005 respectively. *NS*—no significant difference.

Mitochondrial and glycolysis stress tests were performed to assess bioenergetic function of MSCs obtained from 29 of the 39 ELBW infants enrolled in the study as sufficient numbers of MSCs to perform bioenergetic analyses could not be obtained from the remaining 10 infants. Demographic characteristics of these infants grouped by BPD status are available in Supplemental Table E1. Basal and maximal oxygen consumption rates (OCR), ATP-linked OCR and proton leak, spare respiratory capacity (SRC) and ETC complex-IV activity were lower in MSCs from ELBW infants who died or developed moderate/severe BPD versus MSCs from infants who survived with no/mild BPD (Fig. 1 and Table 1). No differences were noted in MSC non-mitochondrial OCR between the 2 groups of infants.

MSCs obtained from term infants (n = 10) who were not at risk for BPD had higher basal OCR as well as ATP-linked OCR compared to MSCs from infants who survived with no/mild BPD as well as those who died or developed moderate/severe BPD. Of note, MSCs obtained from term infants and those from ELBW infants who survived with no/mild BPD had similar maximal OCR as well as proton-leak linked OCR (both were noted to be lower in ELBW infants with moderate/severe BPD). Non-mitochondrial OCR was similar between all 3 groups of infants (Supplemental Figure E1). However, both GA and BW correlated poorly with MSC basal OCR (0.27, *p* = 0.16 and 0.28, *p* = 0.27 respectively) as well as maximal OCR (0.29, *p* = 0.13 and 0.22, *p* = 0.23 respectively). Additionally, MSCs from infants with IUGR had similar basal and maximal OCR when compared to MSCs from infants without IUGR (median[IQR]: 43[38,123] vs. 45[37,62] pmol/min/3 × 10^4 cells respectively, *p* = 0.8 and 83[80,190] vs. 90[72,126] pmol/min/3 × 10^4 cells respectively, *p* = 0.8 respectively), implying that growth restriction also did not significantly modify MSC OCR in this infant cohort.

A multivariate regression model was constructed using GA, IUGR, maternal chorioamnionitis, preeclampsia and smoking status as well as basal OCR (BW and maximal OCR were excluded to avoid multicollinearity with GA and basal OCR respectively) found that risk for moderate/severe BPD or death was lower in infants exposed to maternal chorioamnionitis and those with higher MSC basal OCR (Supplemental Table E2).

**Figure 2:**
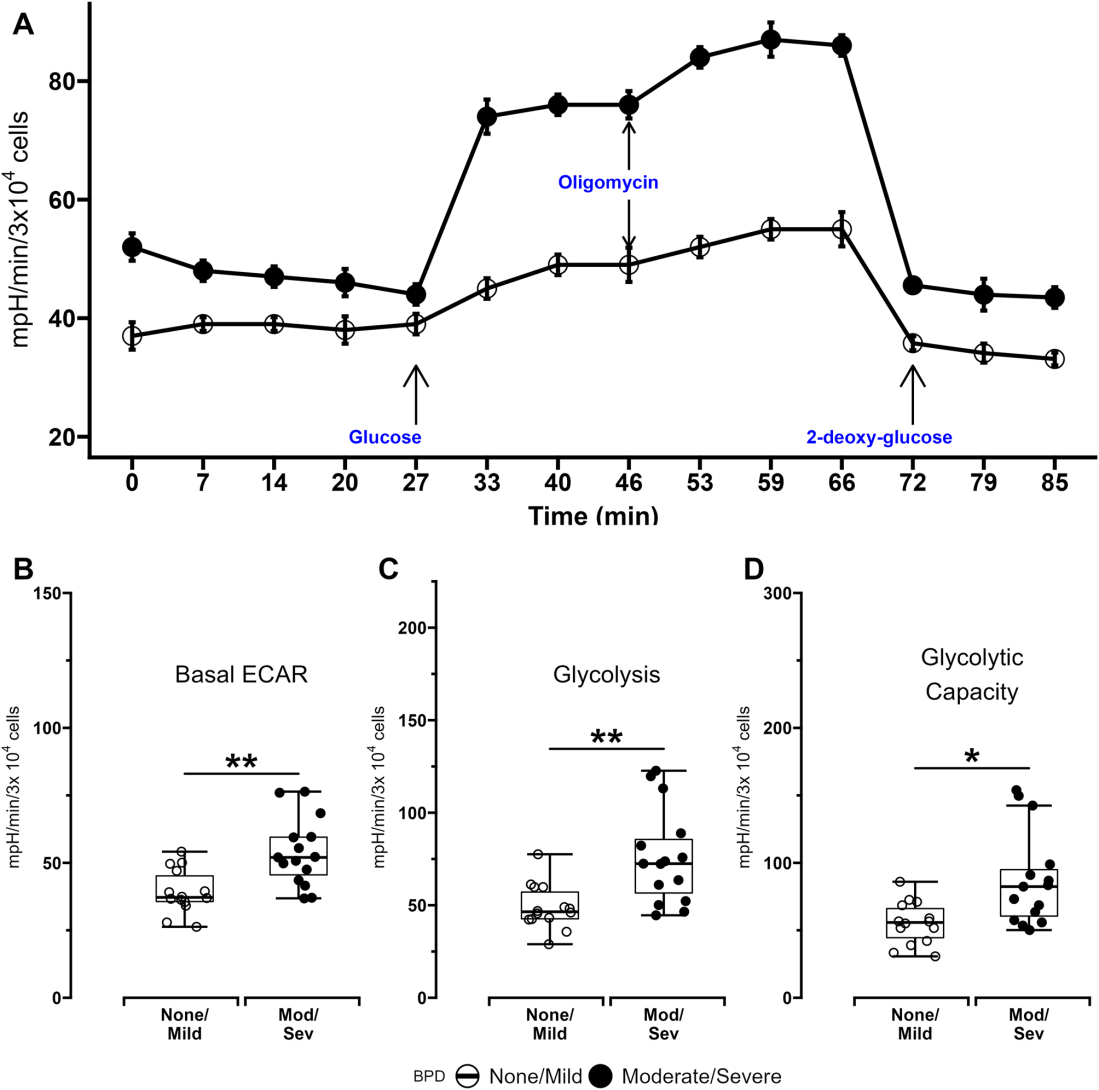
Extracellular acidification rates (ECAR) of MSCs obtained from ELBW infants who died or developed moderate/severe BPD (Mod/Sev) and ELBW infants who survived with no/mild BPD (None/Mild). (**A**) Representative plot of a typical glycolytic stress test conducted using a Seahorse XF96 flux analyzer. 30,000 cells were seeded per well in 96-well plates, and MSC extracellular acidification was measured in the presence of various glycolytic effectors. (**B**) Basal ECAR. (**C**) ECAR during glycolysis (**D**) ECAR due to maximal glycolysis measured after oligomycin introduction. Data for all experiments obtained from MSCs from 15 infants with Mod/Sev BPD and 14 with No/Mild BPD. Differences were analyzed using Mann–Whitney U-test and data expressed as median [25th–75th centiles]. * and ** represent *p* value < 0.05 and < 0.005 respectively. *NS*—no significant difference.

**Figure 3:**
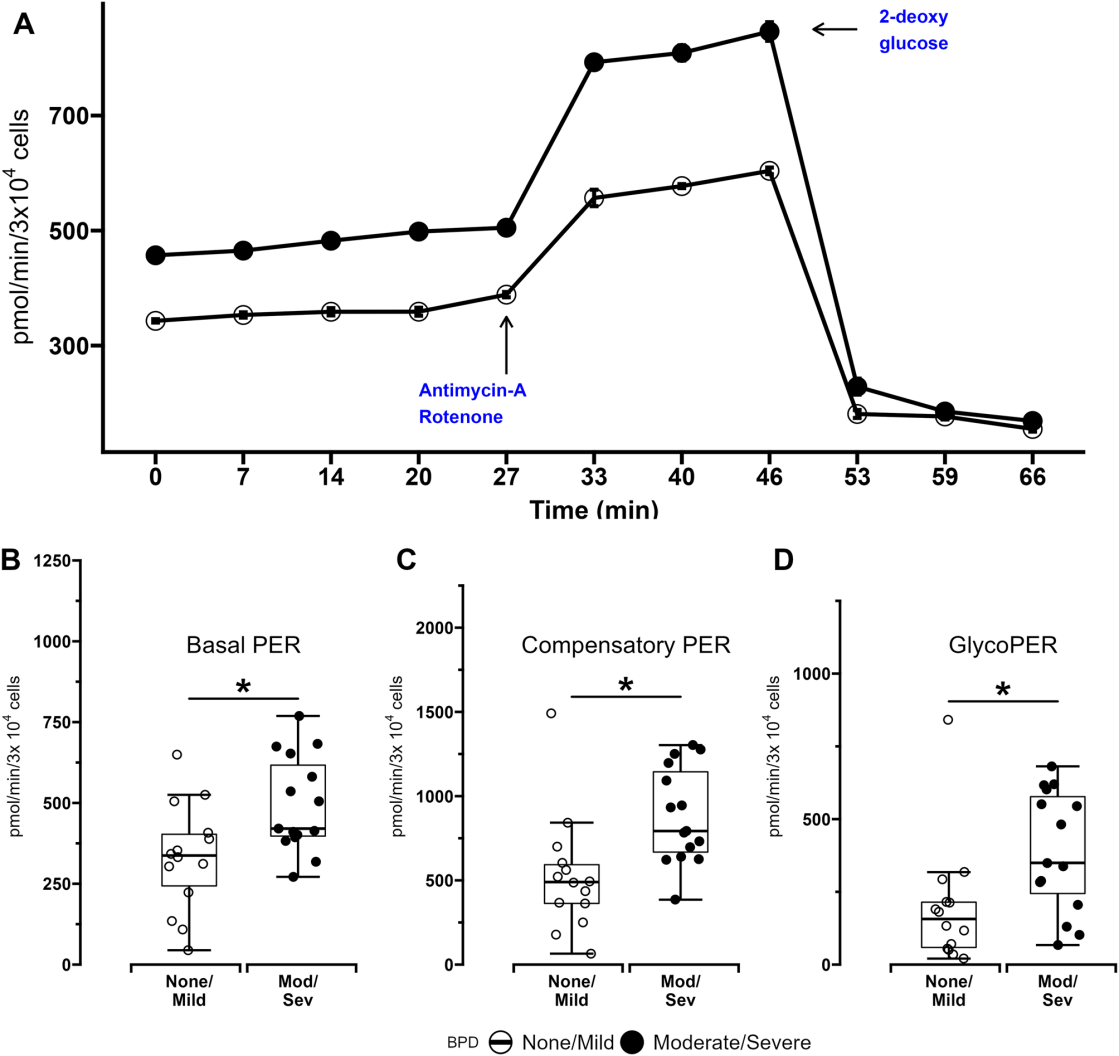
Proton Efflux Rates (ECAR) of MSCs obtained from ELBW infants who died or developed moderate/severe BPD (Mod/Sev) and ELBW infants who survived with no/mild BPD (None/Mild). (**A**) Representative plot of a typical Proton Efflux Rate (PER) Assay conducted using a Seahorse XF96 flux analyzer. 30,000 cells were seeded per well in 96-well plates, and MSC proton efflux was measured at baseline and in the presence of Antimycin-A. 2-deoxy-glucose was used to confirm that proton efflux was specifically related to cellular glycolysis. (**B**) Basal PER (**C**) Compensatory PER due to antimycin A/rotenone-induced inhibition of mitochondrial respiration (**D**) GlycoPER measured as the difference between basal PER and compensatory PER, indicative of glycolysis-specific PER due to maximal glycolysis measured after oligomycin introduction. Data for all experiments obtained from MSCs from 15 infants with Mod/Sev BPD and 14 with No/Mild BPD. Differences were analyzed using Mann–Whitney U-test and data expressed as median [25th–75th centiles]. * and ** represent *p* value < 0.05 and < 0.005 respectively. *NS*—no significant difference.

Quantitative measurement of glycolysis in MSCs was first done using extracellular acidification rates (ECAR). Basal ECAR rates measured in the absence of additional substrates, glycolysis-associated ECAR measured after addition of glucose to the substrate, as well as glycolytic capacity related ECAR measured after inhibition of mitochondrial oxidative phosphorylation using oligomycin were all higher in MSCs from ELBW infants who died or developed BPD compared to MSCs obtained from infants who survived with no or mild BPD (Fig. 2 and Table 1). Proton efflux rates (PER) measured through glycolytic rate assays were also used as an additional measure of glycolysis in MSCs. Higher rates of basal glycolysis and compensatory glycolysis (measured after the addition of mitochondrial inhibitors antimycin-A and rotenone) were again noted in MSCs from infants who died or developed BPD compared to MSCs from infants who survived with no/mild BPD. Glycolysis-specific PER (measured as the difference between PER measured during basal glycolysis and PER measured during compensatory glycolysis) was also higher in MSCs from infants who died or developed BPD compared to infants who survived with no/mild BPD (Fig. 3).

Finally, total cellular ATP content in MSCs grown in standard culture media with glucose and in the absence of any mitochondrial or glycolytic inhibitors was lower in infants who died or developed moderate/severe BPD compared to survivors with no/mild BPD (mean[SD]: 20.23[23.70, 26.70] vs. 29.82[26.81, 30.21] pmol/cell respectively, *p* < 0.001).

### Mitophagy is decreased in MSC from ELBW infants with moderate or severe BPD

**Figure 4:**
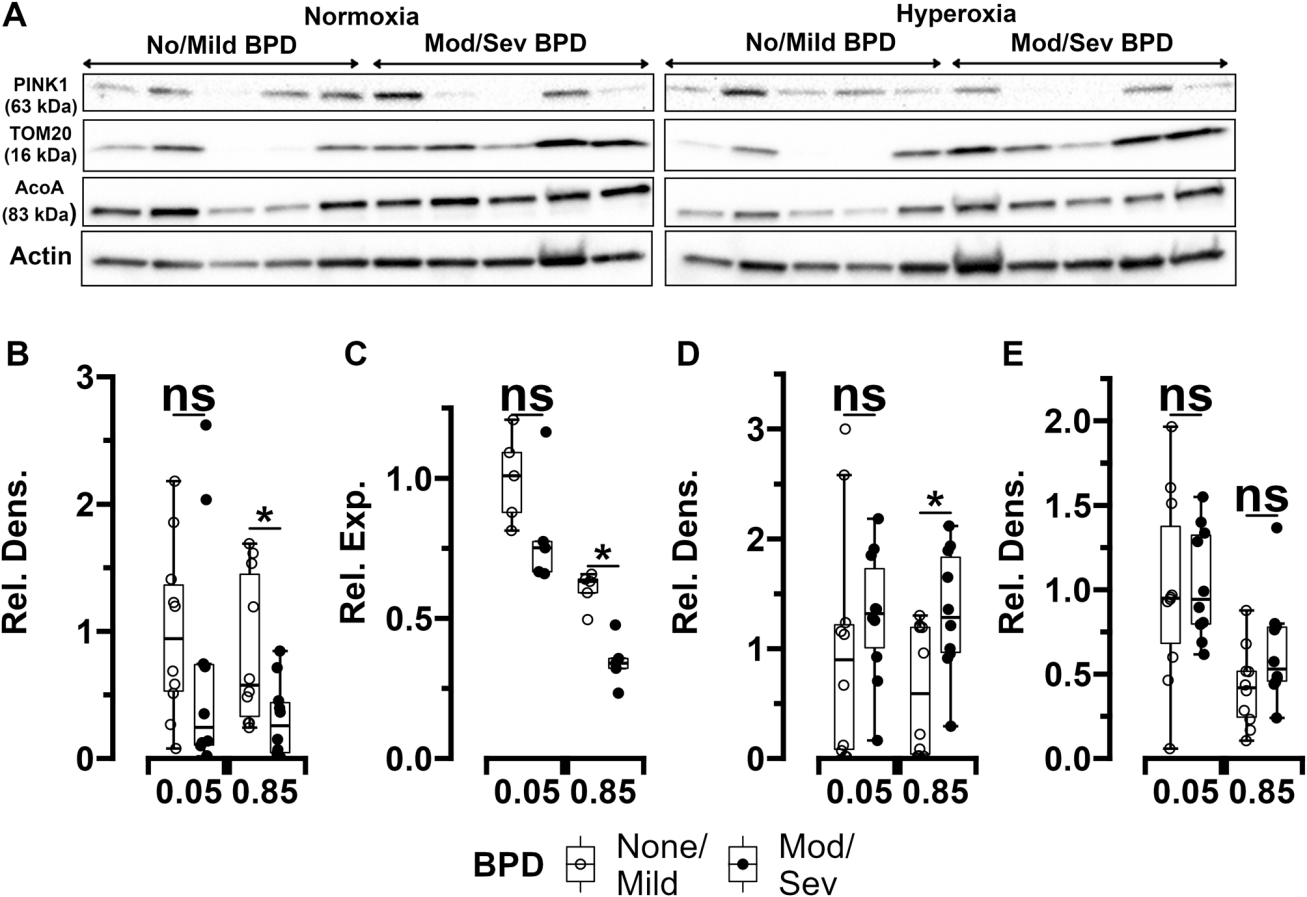
Expression changes of PTEN-induced putative kinase 1 (PINK1), translocase of outer membrane (TOM20), and mitochondrial aconitase (ACO) in MSCs from ELBW infants exposed to normoxia (5%) or hyperoxia (85%) for 24 h. (**A**) Representative Western blot images of ACO, PINK1 and TOM20 in MSCs from infants who survived with no/mild BPD and MSCs who died or developed moderate/severe BPD (n = 5 per group). (**B**) and (**C**) Quantitative densitometric and qPCR analysis of MSC PINK1 protein content (n = 10 per group) and RNA expression (n = 5 per group) respectively. (**D**) and (**E**) Quantitative densitometric analysis of TOM20 and ACO protein content in MSCs (n = 10 per group). Differences were analyzed using Mann–Whitney U-test and data expressed as median [25th–75th centiles]. * and ** represent *p* value < 0.05 and < 0.005 respectively. Rel. Dens.—relative density, Rel. Exp.—relative expression, *NS*—no significant difference. Original gels are presented in Supplemental Figure E5.

MSCs (n = 10 per group) were used to measure expression of the mitophagy mediator PINK1 and the mitochondrial content marker TOM20 (demographic characteristics of these infants grouped by BPD status are available in Supplemental Table E3). Transmission electron microscopy (TEM) images were also obtained (n = 14 per group). When exposed to normoxia (5% O_2_), PINK1 and TOM20 expression were noted to be similar between MSCs obtained from infants who developed moderate/severe BPD and MSCs from infants who survived with no/mild BPD. However, upon exposure to hyperoxia (85% O_2_), PINK1 mRNA and protein levels were lower, while TOM20 protein content was noted to be higher in MSCs obtained from ELBW infants who died or developed moderate/severe BPD compared to MSCs from infants who survived with no/mild BPD (Fig. 4A,D). In addition to using actin as loading control, PINK1 protein expression normalized to mitochondrial content using TOM20 protein expression levels was also higher in normoxia and hyperoxia-exposed MSCs from infants who survived with no/mild BPD compared to MSCs from infants who died or developed moderate/severe BPD (Supplemental Figure E2).

**Figure 5:**
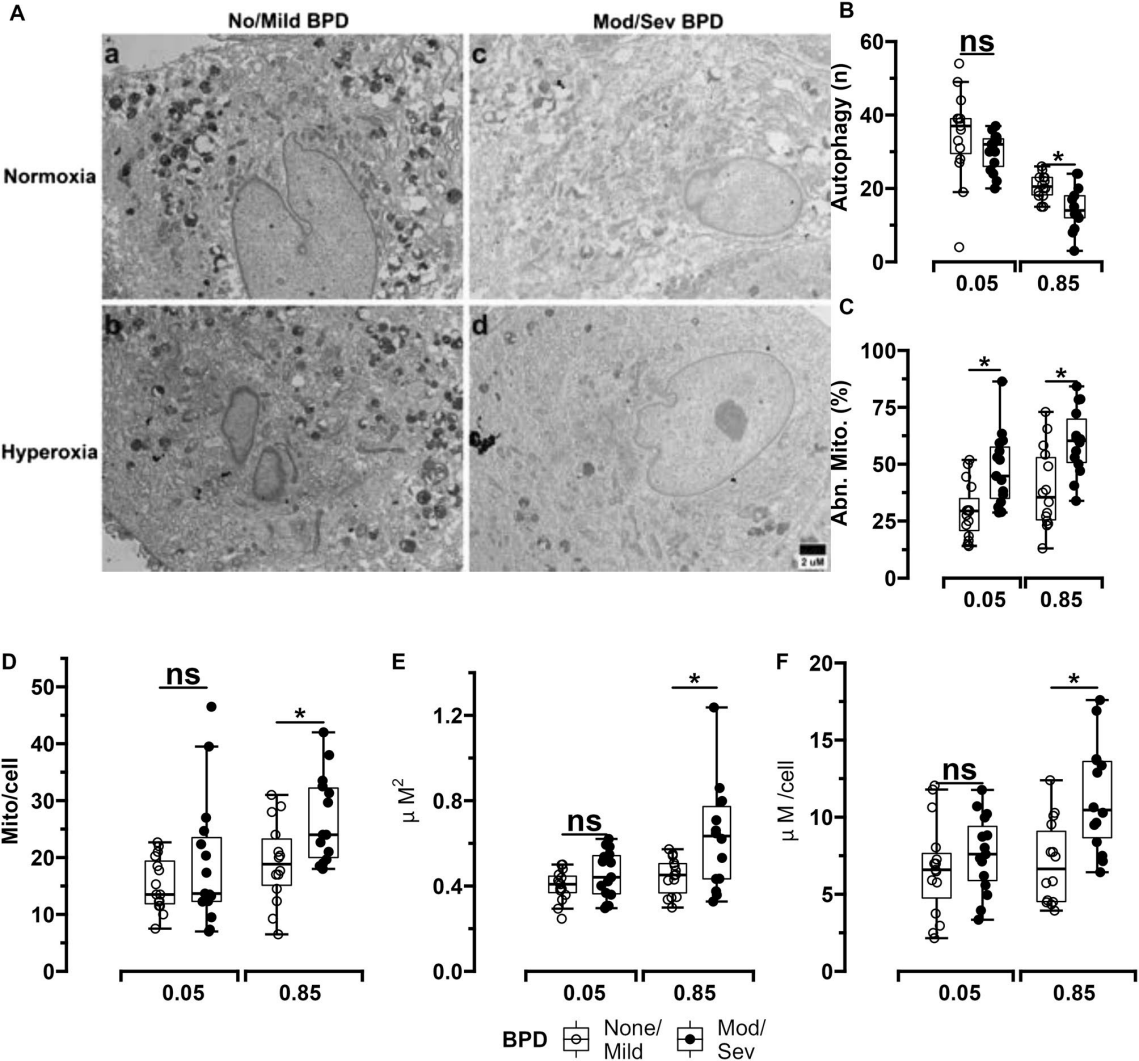
TEM imaging of MSCs from ELBW infants exposed to normoxia (5%) or hyperoxia (85%) for 24 h. (**A**) and (**B**) Representative TEM images and quantification of autophagic vacuoles. MSCs from infants who survived with no/mild BPD exposed to normoxia (a) or hyperoxia (b) had increased number of autophagic vacuoles and less elongated mitochondria versus MSCs from infants who died or developed moderate/severe BPD (c and d). Scale bar represents 2 µm. (**C**) Higher numbers of abnormal mitochondria were found in both normoxia and hyperoxia-exposed MSCs from infants at increased risk for death/moderate/severe BPD versus infants who survived with no/mild BPD. n = 14 per group. Hyperoxia-exposed from MSCs infants with increased BPD risk had increased mitochondrial number per cell (**D**) as well as larger mitochondria (**E**) and consequently increased area occupied by mitochondria per cell (**F**) versus infants who survived without BPD. MSCs from 10 infants per group were used to obtain a minimum of 20 images per infant that were examined by 2 trained independent investigators. Differences were analyzed using Mann–Whitney U-test and data expressed as median [25th–75th centiles]. * and ** represent *p* value < 0.05 and < 0.005 respectively. *NS*—no significant difference.

TEM imaging (Fig. 5A) showed that vacuolar structures—indicative of autophagic foci—were decreased in hyperoxia-exposed MSCs (but not normoxia-exposed MSCs) from infants who died or developed moderate/severe BPD compared to MSCs from survivors with no/mild BPD (Fig. 5B). Mitochondrial morphology was more severely disrupted in both normoxia and hyperoxia-exposed MSCs from infants who died or developed moderate/severe BPD versus MSCs from infants who survived with no/mild BPD (Fig. 5C). Additionally, mitochondria in hyperoxia-exposed MSCs from infants who died or developed moderate/severe BPD were increased in number, larger in size and also occupied a larger area of the cell surface versus mitochondria in hyperoxia-exposed MSCs from infants who survived with mild/no BPD (Fig. 5D,E,F).

### MSC survival is decreased in ELBW infants who developed moderate/severe BPD

**Figure 6:**
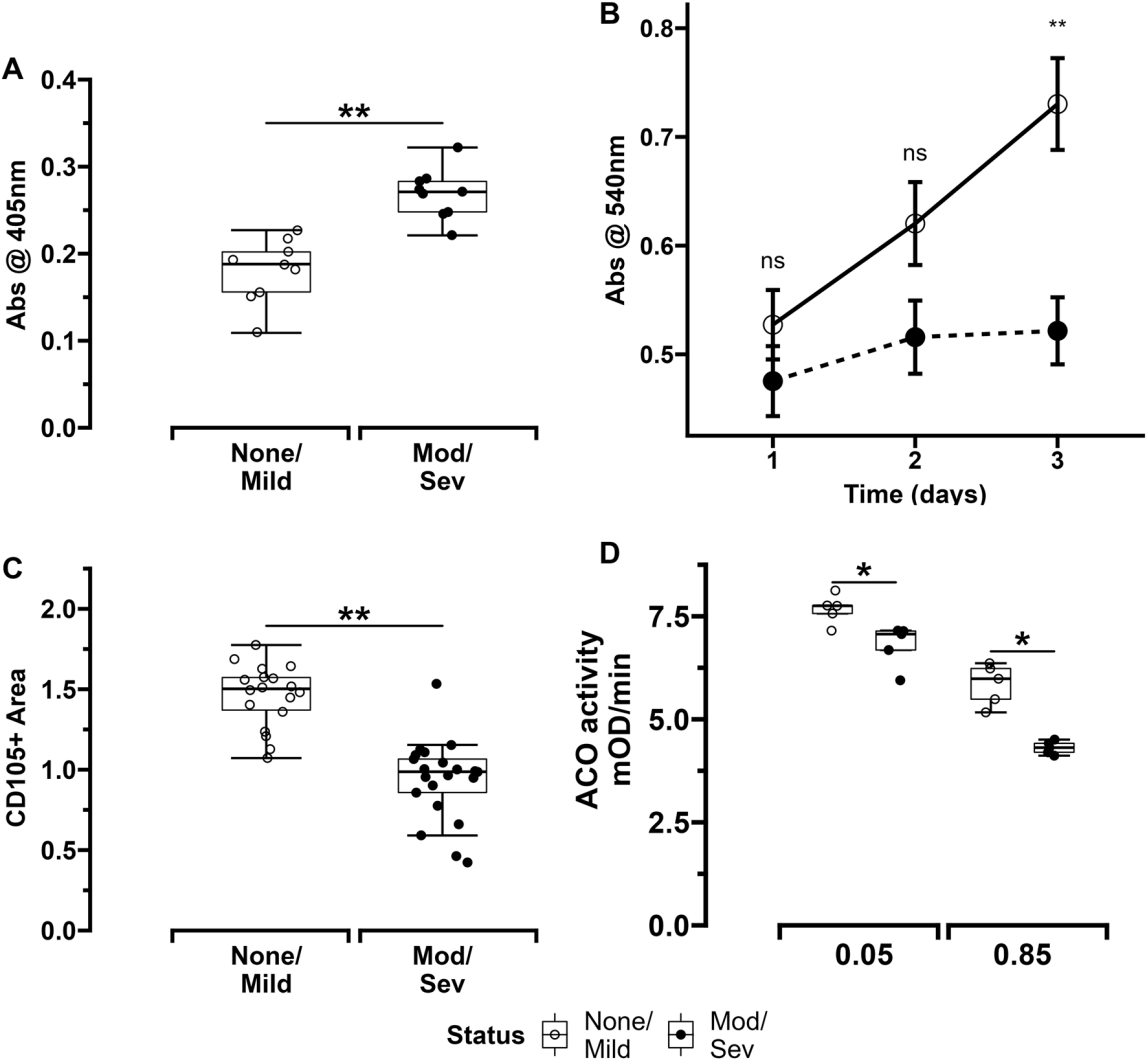
Panel showing MSC survival, proliferation and aconitase activity in ELBW infants. (**A**) MSC apoptosis rates (n = 10 per group). (**B**) MSC proliferation rates measured in cell culture over 3 days (n = 10 per group). (**C**) Quantitative analysis of surface area of umbilical cord sections occupied by MSCs PINK1 protein and RNA expression respectively (n = 10 per group). (**D**) Aconitase activity of MSCs exposed to normoxia (5%) or hyperoxia (85%). n = 5 per group. Differences were analyzed using Mann–Whitney U-test and data expressed as median [25th–75th centiles]. * and ** represent *p* value < 0.05 and < 0.005 respectively. *NS*—no significant difference.

**Figure 7:**
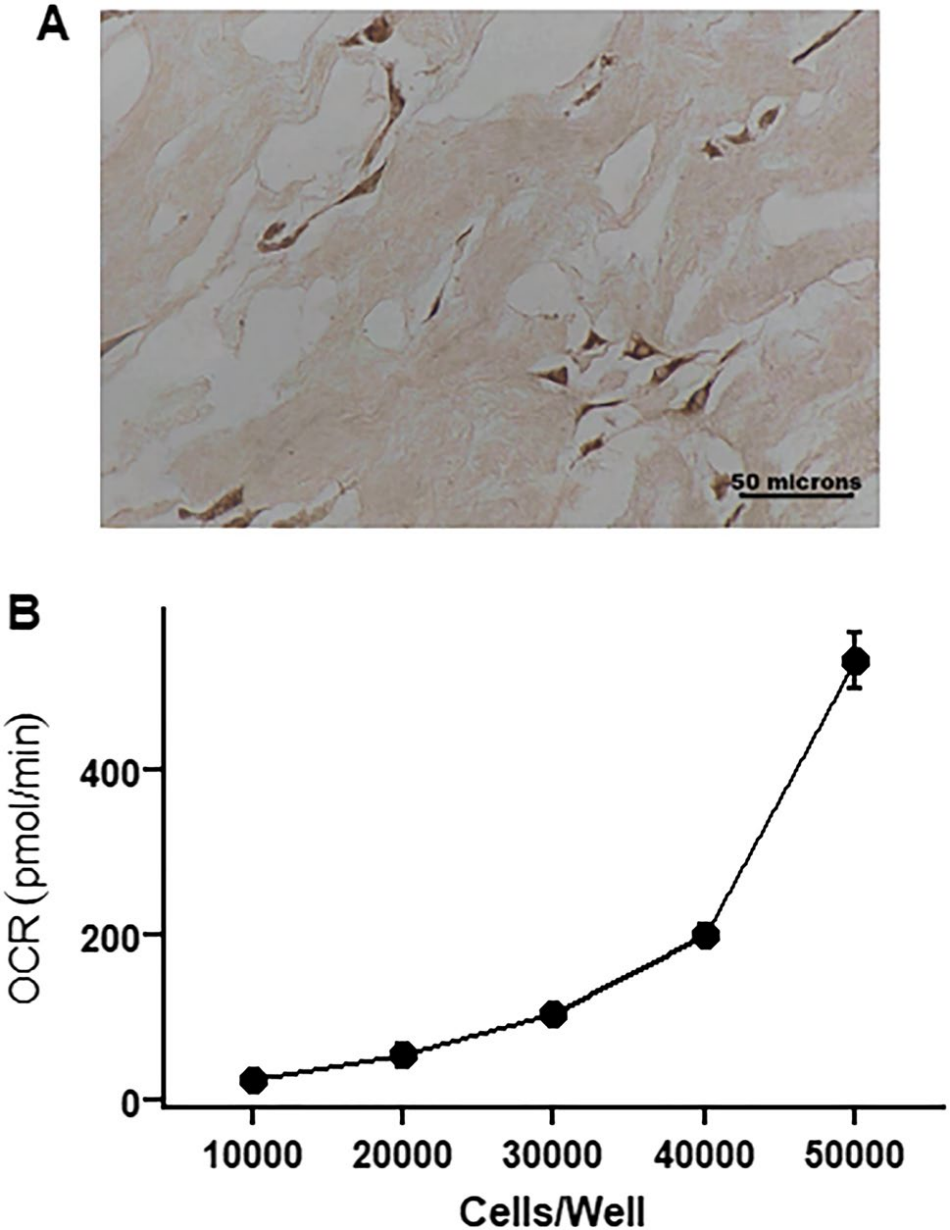
MSC characterization. (**A**) MSCs in umbilical cord tissue sections stained for CD105 antigen. (**B**) Basal oxygen consumption of MSCs seeded at different densities. *MSCs*—mesenchymal stem cells. *OCR*—oxygen consumption rate.

MSCs obtained from infants who died or developed moderate/severe BPD had higher rates of apoptosis versus MSCs from infants who survived with no/mild BPD (Fig. 6A). Proliferation rates of MSCs grown in culture were similar between both groups on days 1 and 2 but found to be lower in infants who died or developed moderate/severe BPD versus infants who survived with no/mild BPD (Fig. 6B). This decreased in-vitro survival of MSCs from infants with increased risk for moderate/severe BPD was also reflected in immunohistochemistry (IHC) images of ex-vivo sections of umbilical cords that showed that MSCs from these infants occupied a relatively lower surface area of cord segments versus those from infants who survived with no/mild BPD (Figs. 6C and 7A).

### MSC oxidant stress is increased in ELBW infants with moderate/severe BPD

Mitochondrial aconitase is known to be a sensitive and critical target of oxidant stress. Aconitase activity also protects against oxidant stress. Therefore, aconitase content and activity in MSCs obtained from ELBW infants were examined. Both hyperoxia and normoxia exposed MSCs from infants who died or developed moderate/severe BPD had similar aconitase protein content versus MSCs from infants who survived with no/mild BPD (Fig. 4A,E). However, aconitase activity was noted to be lower in both hyperoxia and normoxia exposed MSCs from infants who died or developed moderate/severe BPD versus MSCs from infants who survived with no/mild BPD. Additionally, as would be expected, hyperoxia exposure decreased aconitase activity in MSCs from both groups of infants (Fig. 6D). These findings were also associated with higher mtDNA damage in both normoxia and hyperoxia-exposed MSCs from infants who died or developed moderate/severe BPD compared to MSCs from infants who survived with no/mild BPD (Supplemental Figure E3).

## Discussion

Quiescent MSCs typically reside in hypoxic environments in which they are exposed to minimal oxidant stress and fulfill their ATP demands primarily through glycolysis^21–23^. A metabolic switch from glycolysis to oxidative phosphorylation is required to provide both a significant portion of the ATP demand as well as the basal level of ROS stimulation necessary to induce and maintain MSC proliferation and subsequent differentiation into cell lineages that can replace and repair host tissue damage^24–27^. Mesenchymal-epithelial interactions are critical for normal pulmonary alveolarization and MSCs have been found to contribute to lung injury repair by differentiating into alveolar epithelial cells^28–30^. The current study indicates that MSCs obtained at birth from umbilical cords of ELBW infants who later died or developed moderate/severe BPD had lower basal and maximal OCR but increased ECAR and PER compared to MSCs from infants who survived to hospital discharge with no or mild BPD who had oxygen consumption similar to MSCs from normal term infants. Basal OCR was also found to be a significant risk factor for moderate/severe BPD or death in a logistic regression model. The lower oxygen consumption and higher glycolytic activity as well as decreased in-vitro growth and survival in cell cultures noted in umbilical cord-derived MSCs from infants at high risk for BPD suggests that similar mitochondrial dysfunction and decreased proliferation of resident MSCs in the developing lung could contribute to the increased risk for BPD observed in these infants. Furthermore, total basal ATP content in MSCs from infants at risk for severe BPD was also decreased when compared to MSCs from infants at low risk, implying that the increase in glycolysis observed in these MSCs may provide only insufficient compensation for the decline in their mitochondrial bioenergetic function.

Physiological ROS upregulation is also considered essential for activating MSC differentiation^6,31,32^. However, excessive mitochondrial ROS generation, which can be induced in MSCs even during physiologic normoxia (21% O_2_), has been noted to decrease their proliferation, increase apoptosis and reduce engraftment when administered to treat acute respiratory distress syndrome^33^. Mild uncoupling of oxidation from phosphorylation (which lowers mitochondrial membrane potential but does not significantly affect ATP generation) as well as higher spare mitochondrial bioenergetic capacity can protect cells against such oxidant stress related decrease in lifespan^34,35^. Therefore, the lower uncoupled OCR, SRC, aconitase activity, tissue density in umbilical cord sections and increased apoptosis in cell cultures noted in MSCs from infants at high risk for BPD further suggest that mitochondrial dysfunction could lead to reduced MSC lifespan and increased susceptibility to BPD. While aconitase protein content remained similar, aconitase activity as well as mtDNA damage were found to be lower in MSCs from infants who died or developed moderate/severe BPD compared to MSCs from infants who survived with no/mild BPD, implying that differences in post-translational modifications of aconitase, that have been previously noted to be especially relevant under conditions of oxidant stress, may also play a role in the pathogenesis of the BPD^36,37^.

Recent evidence also indicates that MSCs are able to recognize, engulf and degrade damage associated molecular patterns including dysfunctional mitochondria released by neighboring cells, and transfer their own functional mitochondria to such cells^38^. Upon uptake, these MSC-derived mitochondria are able to improve membrane potential, oxidative phosphorylation and redox function of recipient cell mitochondrial networks^16,39–42^. Additionally, EVs released by MSCs from infants have been shown to contain mitochondria as well as functioning isolated mitochondrial ETC complexes that generate the ATP required for cytoskeletal rearrangements through which EV entry into target cell cytoplasm is mediated^43,44^. In this context, the decreased oxygen consumption and complex IV activity identified in MSCs from infants at increased risk for BPD in our study provides a rationale to hypothesize that improving MSC mitochondrial function and activity of ETC complexes could enhance the therapeutic ability of autologous MSC, MSC-derived EVs and conditioned media administration that are currently undergoing clinical trials in ELBW infants at risk for BPD.

Finally, PINK1-mediated mitophagy removes dysfunctional mitochondria and improves cellular bioenergetic and redox health. MSCs deficient in autophagy proteins such as microtubule–associated protein 1 light chain 3B (LC3B) experience significant mitochondrial dysfunction when exposed to oxidant stress^11,45^. Rescuing mitophagy and autophagy or decreasing mitochondrial oxidant stress were both effective in decreasing mitochondrial dysfunction and increasing survival of these cells and upregulation of mitophagy in MSCs that are used in therapeutic transfusions has been shown to improve their therapeutic ability^46,47^. Our findings that lower autophagy and PINK1 expression are associated with higher numbers of mitochondria with ultrastructural and functional abnormalities per cell and TOM20 protein content in MSCs from infants at risk for severe BPD that were exposed to hyperoxia (but not normoxia) indicate that augmenting MSC mitochondrial quality control mechanisms during oxidant stress could also decrease BPD risk.

Limitations of our study include the small sample size of infants included in the study, as well as the heterogeneity in mitochondrial function that is characteristic of cells obtained from different sources which makes extrapolation of umbilical cord-derived MSC bioenergetic changes to bone marrow-derived or resident lung MSCs challenging^48^. However, the umbilical cord-derived exogenous MSC transfusions have been found to decrease neonatal hyperoxic injury in small studies^49–52^. Therefore, the association between mitochondrial dysfunction, decreased autophagy and survival identified in UC-MSCs through this study may be of relevance to help examine the effectiveness of these cells in reducing BPD risk in ELBW infants^53^. Additionally, while in-vitro testing does not permit complete assessment of MSC biology in its physiological niche, MSCs were grown at 5% O_2_ instead of 21% O_2_ to mimic the relatively hypoxic environment that these cells typically experience in-vivo*.* Our study also has several strengths. We used a high throughput bioenergetic assay for both OCR and ECAR to derive a comprehensive profile of bioenergetic function of MSCs obtained from ELBW infants and correlated these measures to their BPD status. Furthermore, the use of intact MSCs allowed us to avoid artefacts often associated with isolated mitochondrial testing^54^. We also note that GA and BW correlated poorly with MSC bioenergetics in this study. However, a previous study found that umbilical cord-derived MSCs from infants born moderately preterm had predominantly glycolytic metabolism whereas MSCs obtained from term infants had more oxidative metabolism^55^. Possible explanations for these differences include methodology differences (OCR was measured using intact cells in a Seahorse™ flux analyzer in this study versus permeabilized cells in an Oxygraph™ in the previous study) as well as the extreme prematurity of the infant cohort included in the current study.

In summary, the findings of our study indicate that umbilical cord-derived endogenous MSC mitochondrial dysfunction in ELBW infants is associated with autophagy/mitophagy deficits, decreased proliferation, and survival as well as increased risk for severe BPD in ELBW infants. Further studies to confirm these findings and to determine the mechanisms responsible for MSC mitochondrial dysfunction in ELBW infants could provide important insights regarding the role of endogenous MSCs and the therapeutic efficacy of autologous MSC transfusions in neonatal lung injury and BPD in ELBW infants.

## Methods

### MSC collection and characterization

Umbilical cord segments immersed in phosphate-buffered saline (Lonza, MD) were minced into 1–2 mm^3^ fragments and aligned at regular intervals in 10 cm culture dishes until they were firmly attached to the bottom of the dish for at least 30 min and expanded in Dulbecco’s modified essential medium (DMEM) supplemented with 10% fetal bovine serum at 37 °C and 5% O2 and 5% CO2. Culture media was replaced 2–3 days after initial plating and then every 2 days. Once the fibroblast-like adherent cells reached 80–90% confluence they were removed from the culture dishes using 10% trypsin and passaged further or cryopreserved for future use.

Adherent cells were characterized as MSCs by following International Society for Cellular Therapy guidelines^56^. An LSRII flow cytometer (BD Biosciences, NJ) and a human MSC verification multicolor flow kit (R&D Systems, MN) were used according to manufacturer instructions to confirm enriched expression of CD105, CD90 and CD73 and decreased expression of the hematopoietic markers CD45, CD34, CD11b, CD79A, HLA-DR. Trilineage differentiation ability of these cells was confirmed by inducing cells plated on coverslips placed in 6 well-plates to differentiate for up to 28 days in basal media to which adipogenic, osteogenic and chondrogenic supplements (R&D Systems, MN) were added, followed by staining for lipids with 0.3% Sudan black, calcium with 2% Alizarin red and glycosaminoglycans with 1% Alcian blue (Millipore Sigma, VT) to confirm their differentiation into adipocytes, osteocytes and chondrocytes respectively (Supplemental Figure E4).

### OCR assay

Preliminary studies of MSC bioenergetics were used to determine that a seeding density of 30,000 MSCs/well produced optimal (100 pmol/min) basal OCR rates best suited for performing mitochondrial stress tests (Fig. 7B). Therefore, 30,000 MSCs were seeded per well into Seahorse™ XF96 Cell Culture Microplates (Agilent) and allowed to adhere for up to 24 h on the day before the assay. On the day of the assay, MSCs were washed once and incubated in DMEM supplemented with 10 mM glucose and 2 mM L-glutamine. After calibration of the Seahorse XF96 Analyzer, the Seahorse™ XF96 Cell Culture Microplate was inserted and basal oxygen consumption rates (OCR) were determined followed by further OCR measurements after the addition of mitochondrial inhibitors Oligomycin (1 µM), carbonyl cyanide-p-triflouromethoxyphenylhydrazone (FCCP, 1 µM), rotenone/antimycin ( 0.5 µM/1 µM) and N,N,N′,N′-tetramethyl-p-phenylenediamine (TMPD)/ascorbic acid (0.5 mM/2 mM) as described previously^8,57^.

### ECAR assay

ECAR, measured using a Seahorse™ XF96 analyzer, was used as a surrogate for anaerobic glycolysis. 30,000 MSCs were seeded per well in Seahorse™ XF96 Cell Culture Microplates (Agilent) and allowed to adhere for up to 24 h on the day before the assay. On the day of the assay, cells were washed with XF base medium and incubated for 60 min at 37 °C in Seahorse XF Base medium supplemented with 5 mM glucose, 2 mM glutamine, 1 mM sodium pyruvate, and 0.5 mM HEPES buffer, pH 7.4 in the absence of CO_2_. Basal ECAR was first measured followed by addition of glucose (10 mM) to measure glycolysis-associated ECAR, oligomycin (2 µM) to inhibit mitochondrial oxidative phosphorylation to measure ECAR associated with maximal glycolysis (glycolytic capacity) and 2-deoxyglucose (100 mM) to confirm suppression of ECAR measurements due to inhibition of glycolysis, as described previously^58^.

### PER assay

As bulk acidification of extracellular medium measured by ECAR are not specific to glycolysis but also includes acidification caused by CO_2_ released through mitochondrial phosphorylation, glycolytic rate assays were also performed to measure glycolysis-specific PER. 30,000 MSCs were seeded per well in Seahorse™ XF96 Cell Culture Microplates (Agilent) and incubated in Seahorse XF Base medium supplemented with 5 mM glucose, 2 mM glutamine, 1 mM sodium pyruvate. Followed by basal measurements of PER, MSCs were next treated with simultaneous addition of antimycin A and rotenone (0.5 µM/1 µM) to inhibit mitochondrial oxidative phosphorylation and subsequent production of carbonic acid produced from the CO_2_ generated by the TCA cycle. PER measurements during this phase enable determination of the mitoPER (PER from mitochondrial respiration) and compensatory glycoPER (PER from glycolysis). Finally, 2-deoxyglucose (100 mM) was injected to inhibit glycolysis and provide qualitative confirmation that proton efflux measured in the experiment was secondary to glycolysis^59^.

### ATP assay

A luminescent ATP Detection Assay Kit (ab113849, Abcam, MA) was used to measure MSC ATP. Briefly, luciferase enzyme and luciferin were added to MSC lysates, and the luminescence emitted was measured using a SpectraMax™ i3 reader (Molecular Devices, CA) and quantified using standard curves for ATP generated by following manufacturer’s instructions.

### Quantitative PCR

RNA from cultured cells exposed to normoxia (5%) or hyperoxia (85%) for 24 h was isolated with RNeasy Plus Mini Kit (Qiagen, Cat. No.74136). The concentration and purity of the RNA were measured checked using SpectraMax i3x (Molecular Devices). Then a total of 1 µg of RNA was reverse-transcribed to cDNA using iScript Reverse Transcription Superrmix (Bio-Rad, Cat. No.1708840), following the manufacturer’s instructions and the cDNA was used as a template in the subsequent qPCR which was performed using TaqMan Fast Advanced Master Mix (Thermo Fisher Scientific. Cat. No. 4444556) with TaqMan gene expression assays for PINK1 (Hs00260868_m1) according to the manufacturer’s instruction of Taqman Fast Advanced Master Mix on the Bio-Rad CFX96 system^60^. All qPCR were carried out using five independent samples in duplicate. To correct the differences in the amount of cDNA loading into qPCR reaction wells, Eukaryotic 18S rRNA Endogenous Control (Thermo Fisher Scientific. Cat. No. 4310893E) was used to normalize the expression levels of the target genes. qPCR data was analyzed by applying the comparative Ct method (ΔΔCT). The results were presented as the fold change in mRNA expression for targeted genes relative to controls.

### Western blotting

MSCs exposed to normoxia (5%) or hyperoxia (85%) for 24 h were lysed in RIPA buffer (Alfa Aesar. Cat. No. J62885) and the lysate was centrifuged at 6,000 g for 10 min at 4ºC, then the supernatant was mixed with 4 × Laemmli sample buffer for western blotting according to standard procedures. 30 to 60 µg of total protein from the samples were separated on 4–20% Criterion TGX Gels (Bio-Rad, Cat. No. 5678093), transferred to PVDF membranes (Trans-Blot Turbo System, Bio-Rad) and probed with antibodies against PINK1 (ab23707), Aconitase (ab181153),TOM20 (ab56783, Abcam, MA) and B-Actin (Cell Signaling Technology, 5125S) as loading control^61^. Membranes were developed using Clarity ECL Substrate (Bio-Rad) and visualized with ChemiDoc imaging System. Densitometry analysis of band intensities was performed using ImageJ software.

### TEM imaging

MSCs exposed to normoxia (5%) or hyperoxia (85%) for 24 h were detached from plates using 0.25% Trypsin and spun down at 1,000 g for 5 min to remove media. The cell pellet was fixed in 0.1 M Sodium Cacodylate Buffer pH 7.4 with 1% Osmium tetroxide (EMS) for 1 h at RT, then dehydrated through a series of graded ethyl alcohols from 50 to 100%. After the infiltration process in propylene oxide, the specimens were embedded in a fresh 100% embedding media and polymerized at 60ºC overnight. For thin sections, the appropriate blocks are cut using a diamond knife (Diatome, Electron Microscopy Sciences, Hatfield, PA) at 70–90 nm (silver to pale gold using color interference) and sections were then placed on copper grids. After drying, the sections were stained with the heavy metals uranyl acetate and lead citrate for contrast and then viewed on a Tecnai Spirit 120kv TEM (FEI, Hillsboro, OR). Digital images were taken with an AMT BioSprint 29^62^.

### IHC of umbilical cord sections

IHC was performed on 10% formalin—fixed and paraffin—embedded (FFPE) 5 µm sections of umbilical cords. Tissue sections on the slides were deparaffinized first with xylene and then rehydrated with alcohol in a graded fashion. Endogenous peroxidases were blocked with 3% hydrogen peroxide and antigen was retrieved by placing the slides in a thermostatic bath of sodium citrate buffer solution for 15–30 min at 98 °C and then allowing them to cool down to room temperature. The tissue sections were then treated with 1% BSA for an hour to block non-specific endogenous antibodies and next with anti CD-105 rabbit monoclonal antibody (Abcam, CA) at 1: 200 dilution after which they were left overnight at 4 °C. After rinsing the antibody incubated tissue sections, the antigen antibody complexes in tissue sections were visualized with diaminobenzidine (DAKO and DAB substrate kit) reaction that resulted in brown staining of the membrane. Images of stained tissue sections were captured using a Nikon-T1E microscope (Nikon, NY). 20 images of umbilical cord sections per infant were analyzed to measure the area of each image occupied by cells positive for stem cell surface markers using ImageJ software.

### Proliferation assay

A CyQUANT™ MTT (3-(4,5-dimethylthiazol-2-yl)-2,5-diphenyltetrazolium bromide) Assay Kit (Thermofisher, MA) was used to measure MSC proliferation. The assay is based on the principle that redox potential of viable mammalian cells causes the conversion of water-soluble MTT to formazan that can be analyzed colorimetrically. 1 × 10^5^ MSCs/well were seeded in 96-well plates and incubated with 100 µL of culture media and 10 µL of 12 mM MTT solution for 4 h. 100 µL of sodium dodecyl sulfate was next added to each well to solubilize the formazan. Each microplate was incubated for a further 12 h at 37 °C and absorbance was measured at 540 nm using a SpectraMax™ reader. 10 µL of the MTT stock solution to 100 µL of medium alone was included as a negative control for each sample^63^.

### Apoptosis assay

Caspase-3 activity in MSC lysates was determined using the Caspase-3 Colorimetric Assay Kit (BioVision, CA).Following manufacturer’s instructions, 5 × 10^6^ MSCs were lysed and centrifuged at 10,000 × g for 1 min and the supernatant, reaction buffer (10 mM DTT) and Asp-Glu-Val-Asp–p-nitroanilide (a substrate for caspase-3) were incubated for 2 h at 37 °C and transferred to 96-well plates. Absorbance at 405 nm was read by a SpectraMax™ reader^64^.

### Aconitase activity

To measure mitochondrial aconitase activity, an aconitase activity kit (Abcam, CA) was used as per manufacturer’s instructions. Briefly, MSCs in six‐well plates were exposed to normoxia or hyperoxia for 24 h. After treatment, cells were homogenized in 100 μL of assay buffer provided and spun at 20,000 g for 15 min at 4 °C to separate mitochondrial fractions. 300 μg of isolated mitochondria per sample were re‐suspended with 100 μL of assay buffer and then incubated for 1 h with the isocitrate substrate mixture at 25 °C and absorbance at 240 nm was recorded for 30 min using a SpectraMax™ reader. Catalytic activity of aconitase was determined by measuring the rate of formation of cis-aconitate as detected by increase in the absorbance^65^.

### MtDNA damage assessment

*G*enomic DNA (gDNA) that was extracted and quantified from MSCs between passages 2–4 (10 per group) that were exposed to normoxia or hyperoxia for 24 h was used to amplify 8.9 kb (forward 5’ TCTAAGCCTCCTTATTCGAGCCGA-3′, reverse 5′-TTTCATCATGCGGAGATGTTGGATGG-3′) and 0.25 kb (forward 5′-CCCCCATAAATAGGAGAAGGCTTAG-3, reverse 5′-TTTCATCATGCGGAGATGTTGGATGG-3′) mtDNA fragments as well as 0.095 kb (forward 5′-GCTGGGTAGCTCTAAACAATGTATTCA-3′, reverse 5′-CCATGTACTAACAAATGTCTAAAATGGT-3′) gDNA fragments which were then quantified as described previously to calculate frequency of mtDNA lesions in these cells^8^.

### Statistical analysis

Student’s t-test (parametric), Wilcoxon rank sum test (non-parametric) and Chi-Square test or Fisher’s exact test (categorical) were used to test for differences between individual pairwise comparisons. Correlation between continuous variables was evaluated using Pearson’s test. All analyses were performed using R 4.0.2^66^. Results are expressed as mean ± SD or median (IQR). *p* < 0.05 was considered significant. To detect a difference of 30 ± 15 pmol/min/30 k cells (mean ± SD) difference in MSC basal OCR (based on OCR measurements in umbilical venous endothelial cells obtained from ELBW infants in our previous study) at 5% level of significance (α = 0.05), and 80% power, we estimated that we would need a sample size of 15 infants per group^8^. A logistic regression model was also constructed to test the strength of association between risk for death or moderate/severe BPD, clinical risk factors and MSC basal OCR.

### Study approvals

All protocols were approved by the Institutional Review Board of the University of Alabama at Birmingham (UAB). Infants were enrolled after appropriate informed consent was obtained from their mothers before or within 6 h after birth. All procedures and experiments conducted in the study were carried out in accordance with all relevant institutional guidelines and regulations.

## Supplementary Information

Supplementary Information.

## Data Availability

The clinical datasets and the various experimental data analyzed to perform the current study are available from the corresponding author on reasonable request.
