# Supplementary material for: Mesenchymal stem cell bioenergetics and apoptosis are associated with risk for bronchopulmonary dysplasia in extremely low birth weight infants
Source: Sci Rep. 2022 Oct 19;12:17484. doi: 10.1038/s41598-022-22478-5 (PMC9582007; doi:10.1038/s41598-022-22478-5)
Supplement: Supplementary file 1 — Supplementary Information. [file 41598_2022_22478_MOESM1_ESM.docx]

#### **ONLINE DATA SUPPLEMENT**

This appendix has been included to provide readers with additional information about the manuscript **Mesenchymal stem cell bioenergetics and apoptosis are associated with risk for bronchopulmonary dysplasia in extremely low birth weight infants**

**Authors**: Snehashis Hazra^1^, Rui Li^1^, Bianca M. Vamesu^1^, Tamas Jilling^1^, Scott W Ballinger^2^, Namasivayam Ambalavanan^1,2^, Jegen Kandasamy^1^

## **FIGURE LEGENDS**

**Figure E1:** **Oxygen consumption rates (OCR) of MSCs obtained from normal term infants compared to ELBW infants who died or developed moderate/severe BPD (Mod/Sev) and ELBW infants who survived with no/mild BPD (None/Mild).** **(A)** Basal OCR. **(B)** Maximal OCR measured after FCCP introduction**. (C)** Panel with plots showing MSC ATP-linked OCR, proton leak, and non-mitochondrial OCR. Data for all experiments obtained from MSCs from 10 term infants, 15 infants with Moderate/Severe BPD and 14 with No/Mild BPD. Differences were analyzed using Mann-Whitney U-test and data expressed as median [25th - 75th centiles]. * and ** represent p-value < 0.05 and < 0.005 respectively. *NS* - no significant difference.

**Figure E2:** **PINK1 expression in MSCs**. PINK1 expression in MSCs exposed to normoxia or hyperoxia normalized for TOM20 protein which is a marker of cellular mitochondrial content. Differences were analyzed using Mann-Whitney U-test and log-transformed values are expressed as median [25th - 75th centiles]. * and ** represent p-value < 0.05 and < 0.005 respectively. n=10 per group.

**Figure E3:** **Mitochondrial DNA damage.** Frequency of mtDNA lesions noted in normoxia and hyperoxia-exposed MSCs from ELBW infants. Differences were analyzed using Mann-Whitney U-test and data expressed as median [25th - 75th centiles]. * and ** represent p-value < 0.05 and < 0.005 respectively. n=10 per group.

**Figure E4:** **MSC characterization through surface antigen expression and trilineage differentiation.** Representative figures of flow cytometry experiments indicate moderate expression of CD105 (**A**) and higher expression of CD73 (**B**) as well as CD90 (**C**) and minimal expression of the hematopoietic markers CD45, CD34, CD11b, CD79A and HLA-DR. Sudan black staining of lipid inclusions (**D**), Alizarin red staining of calcium deposits (**E**) and Alcian blue staining of proteoglycan inclusions (**F**) in adherent cells isolated from umbilical cords of ELBW infants that were induced to differentiate into adipocytes, osteocytes and chondrocytes.

**Figure E5:** **Western blots.** Original gels of the representative western blot bands presented in Figure 4. All lanes have been labelled to identify the protein of interest, infant group and MSC oxygen exposure.

## **FIGURES**

**Figure E1**


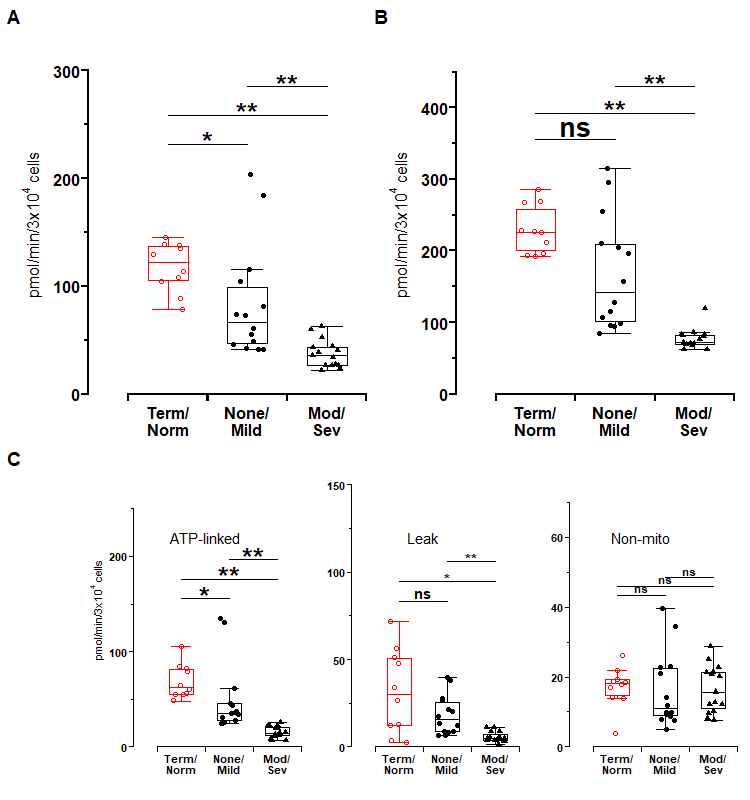

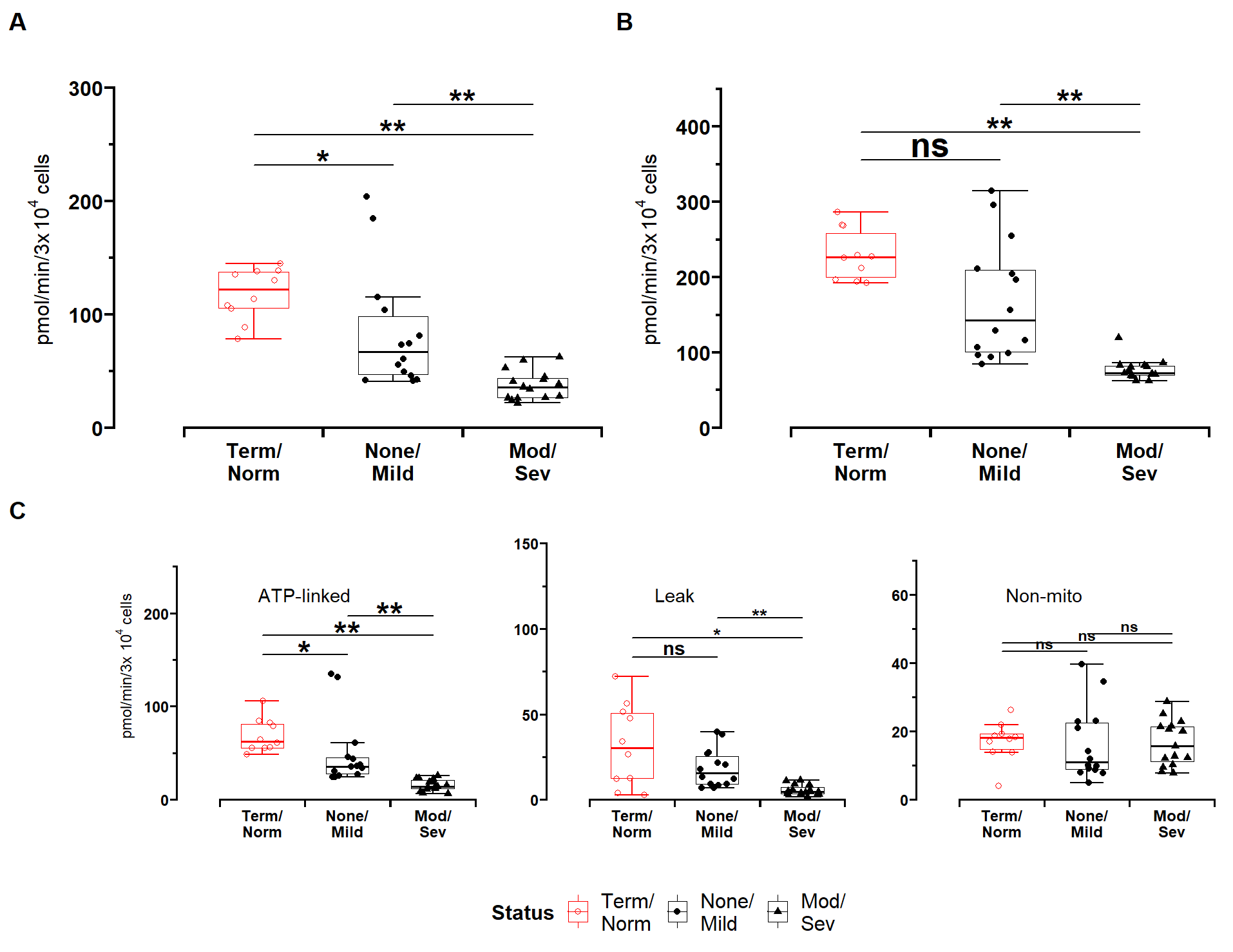


**Figure E2**

**
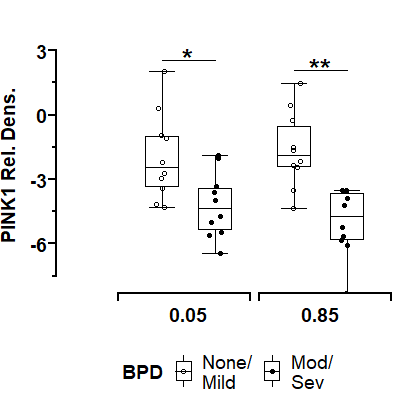
**

**Figure E3**


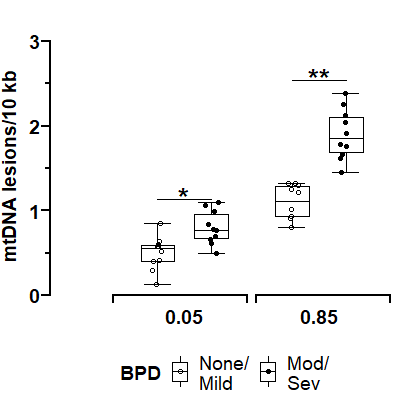


**Figure E4**


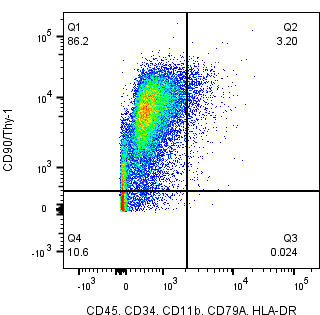

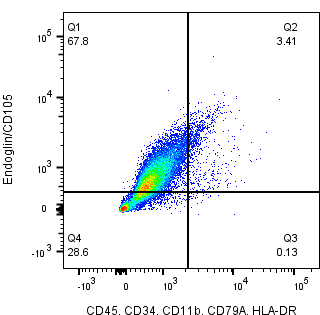


**A**


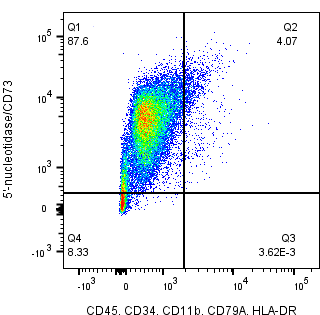


**B**


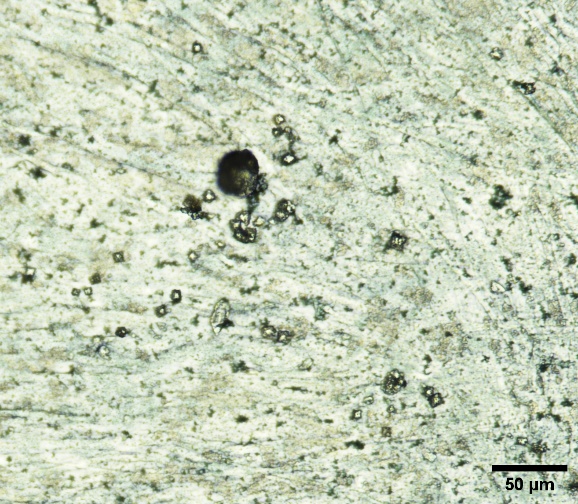


**D**


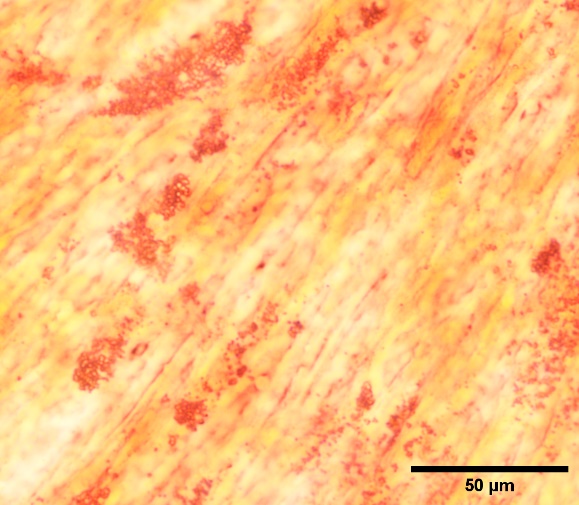


**E**


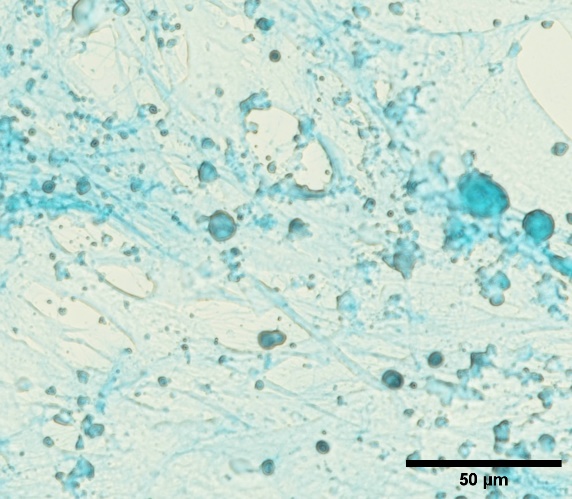


**F**

**C**

**Figure E5**

## **
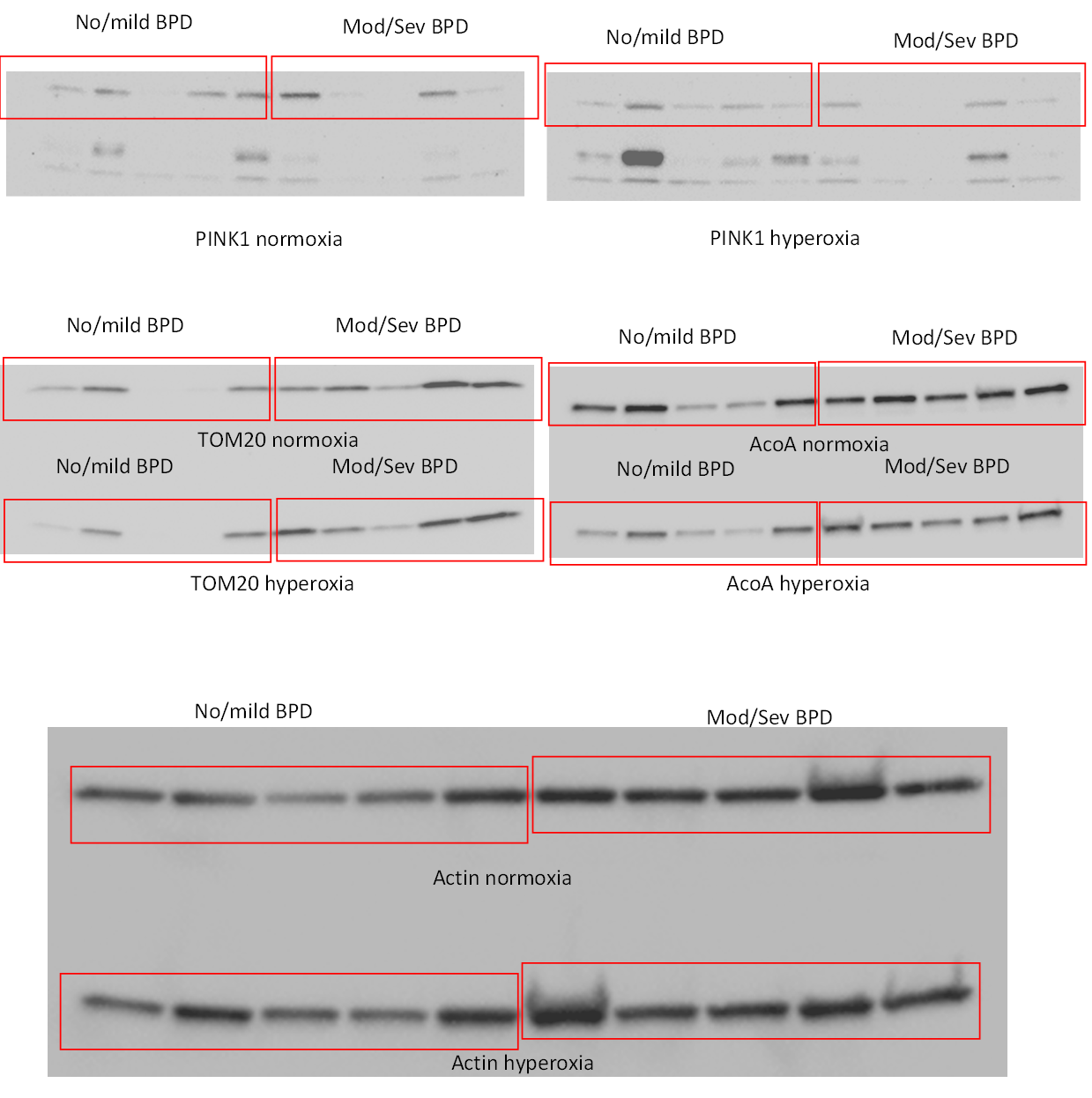
**

## **TABLES**

**Table E1**: Clinical characteristics by BPD Status of infants from whom MSC bioenergetic studies were obtained.

| **Characteristic** | **N** | **No/Mild BPD**  **N = 14^1^** | **Moderate/Severe BPD**  **N = 15^1^** | **p-value^2^** |
| --- | --- | --- | --- | --- |
| **GA (weeks)** | 29 | 26.00 [26.00, 27.00] | 25.00 [24.00, 26.00] | **0.029** |
| **Birth Weight (g)** | 29 | 870 [748, 980] | 720 [636, 942] | 0.10 |
| **Sex (Female)** | 29 | 8 [57%] | 5 [33%] | 0.2 |
| **Intrauterine Growth Restriction** | 29 |  |  | >0.9 |
| N |  | 13 [93%] | 13 [87%] |  |
| Y |  | 1 [7.1%] | 2 [13%] |  |
| **Ethnicity** | 29 |  |  | **0.035** |
| Caucasian |  | 3 [21%] | 9 [60%] |  |
| African-American |  | 11 [79%] | 6 [40%] |  |
| **Histologic Chorioamnionitis** | 29 | 13 [93%] | 2 [13%] | **<0.001** |
| **Preeclampsia/ PIH/ Eclampsia** | 29 | 5 [36%] | 5 [33%] | >0.9 |
| **Maternal Smoking** | 29 | 5 [36%] | 8 [53%] | 0.3 |
| ^1^Median [IQR]; n [%], ^2^Student's t-test; Pearson's Chi-squared test; Wilcoxon rank sum exact test | | | | |

**Table** **E2**: Characteristics of regression model to predict risk for death/moderate/severe BPD

| **Characteristic** | **OR^1^** | **95% CI^1^** | **p-value** |
| --- | --- | --- | --- |
| **GA** | 0.65 | 0.33, 1.27 | 0.2 |
| **IUGR** | 1.12 | 0.02, 65.2 | >0.9 |
| **Preeclampsia** | 1.57 | 0.19, 16.9 | 0.7 |
| **Chorioamnionitis** | 0.01 | 0.00, 0.07 | <0.001** |
| **Smoking** | 2.16 | 0.28, 21.0 | 0.5 |
| **Basal OCR** | 0.40 | 0.14, 0.73 | 0.019* |
| ^1^OR = Odds Ratio, CI = Confidence Interval. * - P < 0.05, ** - P < 0.005 | | | |

**Table E3**: Clinical characteristics by BPD status of infants from whom MSC PINK1, aconitase and TOM20 expression & mtDNA damage analyses were obtained.

| **Characteristic** | **N** | **No/Mild BPD**  **N = 10^1^** | **Moderate/Severe BPD**  **N = 10^1^** | **p-value^2^** |
| --- | --- | --- | --- | --- |
| **GA (weeks)** | 20 | 26.50 [26.00, 27.00] | 25.00 [24.25, 25.00] | **0.011** |
| **Birth Weight (g)** | 20 | 910 [765, 1,108] | 698 [629, 825] | **0.045** |
| **Sex (Female)** | 20 | 7 [70%] | 3 [30%] | 0.074 |
| **Intrauterine Growth Restriction** | 20 |  |  | >0.9 |
| N |  | 9 [90%] | 9 [90%] |  |
| Y |  | 1 [10%] | 1 [10%] |  |
| **Ethnicity** | 20 |  |  | 0.3 |
| Caucasian |  | 2 [20%] | 5 [50%] |  |
| African-American |  | 8 [80%] | 5 [50%] |  |
| **Histologic Chorioamnionitis** | 20 | 6 [60%] | 2 [20%] | 0.2 |
| **Preeclampsia/ PIH/ Eclampsia** | 20 | 3 [30%] | 3 [30%] | >0.9 |
| **Maternal Smoking** | 20 | 3 [30%] | 5 [50%] | 0.6 |
| ^1^Median [IQR]; n [%], ^2^Student's t-test; Pearson's Chi-squared test; Wilcoxon rank sum exact test | | | | |
